# Cortical Damage Associated With Cognitive and Motor Impairment in Hereditary Spastic Paraplegia: Evidence of a Novel *SPAST* Mutation

**DOI:** 10.3389/fneur.2020.00399

**Published:** 2020-05-27

**Authors:** Jian-zhong Lin, Hong-hua Zheng, Qi-lin Ma, Chen Wang, Li-ping Fan, Han-ming Wu, Dan-ni Wang, Jia-xing Zhang, Yi-hong Zhan

**Affiliations:** ^1^Magnetic Resonance Center, The Affiliated Zhongshan Hospital of Xiamen University, Xiamen, China; ^2^Fujian Provincial Key Laboratory of Neurodegenerative Disease and Aging Research, Institute of Neuroscience, School of Medicine, Xiamen University, Xiamen, China; ^3^Department of Neurology, The First Affiliated Hospital of Xiamen University, Xiamen, China; ^4^Institute of Brain Diseases and Cognition, School of Medicine, Xiamen University, Xiamen, China

**Keywords:** *SPAST* gene mutation, SPG4-hereditary spastic paraplegia, MRI, brain, gray-matter changes

## Abstract

To determine the cortical mechanism that underlies the cognitive impairment and motor disability in hereditary spastic paraplegia (HSP), nine HSP patients from a Chinese family were examined using clinical evaluation, cognitive screening, and genetic testing. Controls were matched healthy subjects. White-matter fractional anisotropy (FA), mean diffusivity (MD), axial diffusivity (AD), and radial diffusivity (RD; tract-based spatial statistics), cortical thickness (FreeSurfer), and subcortical gray matter (FIRST) based on T1-weighted MRI and diffusion tensor imaging were analyzed. A novel mutation in the *SPAST* gene (NM_014946.3, c.1321+2T>C) was detected. Patients had motor disability and low Montreal Cognitive Assessment (MoCA) scores. Patients showed significantly decreased total gray- and white-matter volumes, corpus callosum volume, cortical thickness, and subcortical gray-matter volume as well as significantly lower FA and AD values and significantly higher MD and RD values in the corpus callosum and corticospinal tract. Cortical thickness, subcortical gray-matter volume, and MoCA score were negatively correlated with disease duration. Cortical thickness in the right inferior frontal cortex was negatively correlated with Spastic Paraplegia Rating Scale score. Cortical thickness and right hippocampus volume were positively correlated with the MoCA score and subscores. In conclusion, brain damage is not restricted to the white matter in SPG4-HSP patients, and widespread gray-matter damage may account for the disease progression, cognitive impairment, and disease severity in SPG4-HSP.

## Introduction

Hereditary spastic paraplegia (HSP) is a heterogeneous neurodegenerative disorder, with over 70 causative genes (1). Different HSP genotypes exhibit distinct brain features on MRI. One of the most extensively studied HSP genotypes, SPG11-HSP, is associated with corpus callosum thinning on MRI (2–4). SPG15-HSP is also associated with corpus callosum damage (5), while SPG2 (6), and SPG35 (7) are associated with damage to the internal capsule, brainstem, and cerebellum.

SPG4 is the most common genotype of HSP, accounting for almost 50% of HSP families (8). It is linked to abnormal brain function in terms of the cerebral blood flow (9), neuronal biochemistry (10), cortical sensorimotor function (11), and resting-state neuronal activity (12). Moreover, damage has been found in brain structures in the white matter, including the corticospinal tracts, cingulum, cerebral pedicle, internal capsule, cerebellum, and corpus callosum (3, 13–15).

SPG4-HSP is characterized by progressive lower-limb spasticity and is also linked to mental deficits (16, 17). Most studies of SPG4-HSP have revealed an association between white-matter changes in the motor pathways and motor disability. However, the relationship of cortical gray-matter changes with motor disability as well as the brain mechanisms underlying the cognitive impairments in SPG4-HSP remain unclear. We hypothesized that in addition to white-matter damage, SPG4-HSP may be associated with cortical impairment.

In this study, a Chinese family with HSP was recruited. We first performed clinical evaluation and cognitive screening, then determined their HSP genotype, and finally conducted multiple MRI studies on the cortical and subcortical gray matter. Moreover, to enable direct comparisons of our findings with those of previous studies, we analyzed the white-matter changes. We aimed to infer the possible correlations of gray- and white-matter changes with cognitive and motor dysfunction in HSP.

## Materials and Methods

### Subjects

**Figure 1:**
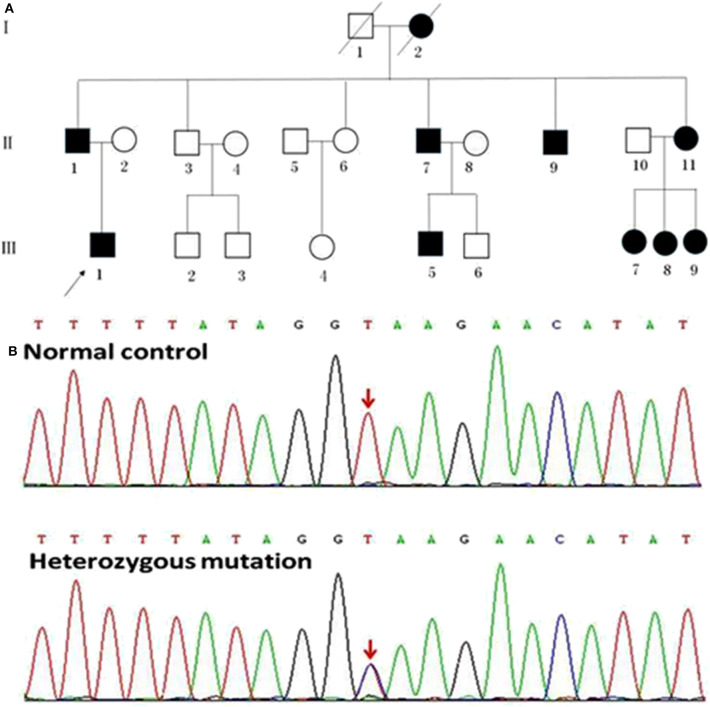
Pedigrees of the HSP patients with *SPAST* splicing mutations. **(A)** The Roman numbers one the left of the pedigrees indicate the generation. The numerals below the patient symbols indicate the patient order. The arrow shows the proband who had the *SPAST* c.1321+2T>C mutation. The black and open symbols show the family members with and without HSP, respectively. The diagonal line indicates the family members who have passed away. **(B)** The sequencing chromatogram of the index patient indicates a heterozygous *SPAST* c.1321+2T>C mutation (nomenclature according to National Center for Biotechnology Information Reference Sequence: NM_014946.3).

We enrolled a Chinese family with HSP undergoing treatment in the First Affiliated Hospital of Xiamen University (Figure 1A; Table 1). Patients' self-reports of their first experience of motor disability were deemed as the age of disease onset. Patients were excluded if they had a history of injury, a documented neurological disorder, coronary artery disease, diabetes, or other diseases known to affect somatic movement and cognition. Patients II 7 and III 5 were unable to attend the MRI scanning because they had metal implants in their body. Thus, only seven patients with confirmed SPG4 mutations (four females, three males; mean age, 44.14 ± 17.10 years) underwent MRI scanning. Seven age- and sex-matched healthy subjects (four females, three males; mean age 45.57 ± 15.71 years) were collected as controls. All subjects were right handed. Informed consent was obtained from the participants or from a proxy prior to enrollment. The study protocol was approved by the Research Committee and the institutional review board of the First Affiliated Hospital of Xiamen University. The Spastic Paraplegia Rating Scale (SPRS) scores of the family members were calculated. The SPRS is scored from 0 to 52, with higher scores indicating greater severity (18).

**Table 1:** Summary of clinical features of the HSP patients.

**Patients**	**II1**	**II7**	**II9**	**II11**	**III1**	**III5**	**III7**	**III8**	**III9**
Age (years)	68	64	60	58	33	32	33	30	27
Gender	M	M	M	F	M	M	F	F	F
Disease duration (years)	13	9	19	18	8	2	10	2	2
Spastic gait	+	–	+	+	+	–	+	–	+
Weakness	+	+	+	+	+	+	+	+	+
Amyotrophy	–	–	+	+	+	–	+	–	–
Knee reflex	3+	3+	4+	4+	4+	3+	4+	3+	3+
Babinski Sign	+	+	+	+	+	–	+	–	+
Ankle clonus	–	–	+	+	+	–	+	–	–
Deep Sensory Loss	–	–	–	–	–	–	–	–	–
Claw foot	+	–	+	+	+	–	+	–	–
SPRS	7	2	32	35	20	3	36	2	3

*Knee reflex 3+, hyperactive without clonus; 4+, hyperactive with clonus; –, negative; +, positive; SPRS, Spastic Paraplegia Rating Scale; HSP, hereditary spastic paraplegia. Patients II 7 and III 5 were unable to undergo MRI scanning because they had metal implants in their body*.

### Cognitive Screening

The Montreal Cognitive Assessment (MoCA) was used as a screening instrument for identifying patients with dementia, and scored as follows (19): orientation (six points), attention (six points), visuospatial and executive functioning (five points), delayed recall (five points), language (three points), animal naming (three points), and abstraction (two points). The total MoCA score ranges from 0 to 30, with scores ≥26 considered normal. All subjects were administered the MoCA by the same experienced neurologists.

### Genetic Examination

Genetic examination was conducted using targeted exome sequencing at Kingmed Diagnostics, Guangzhou, China. DNA was extracted from the blood samples of patients by using QIAamp Blood DNA Mini Kit (Qiagen, Valencia, CA, USA) and sequenced on a NextSeq500 sequencer (Illumina Inc., San Diego, CA, USA) to detect 4,811 clinically relevant genes, including several genes linked to HSP. Candidate mutations in the proband and other family members were identified using conventional Sanger sequencing. Splice-site mutations were analyzed using Human Splicing Finder (HSF3) and Alamut (Interactive Biosoftware).

### Analysis of *SPAST* Transcripts

To experimentally confirm the effect of the c.1321+2T>C mutation on the mRNA transcripts of *SPAST*, we amplified the cDNA synthesized from the leukocytes of patients II 1 and III 1 as well as a healthy control subject without the *SPAST* mutation was amplified with the primers (F: 5′-gatgatatagctggtcaagactt-3′; R: 5′-cttgtctcctcatttggtaaaga-3′) that matched the exons 8–13. The resulting PCR fragments were then purified and sequenced.

### MRI Studies

Brain images were obtained using a Verio 3T scanner (Siemens, Erlangen, Germany) at the MRI Center of Zhongshan Hospital, Xiamen, China. T1-weighted images were scanned with the following parameters: repetition time (TR), 1900 ms; echo time (TE), 2.7 ms; average, 1; field of view (FOV), 25 × 25 cm^2^; matrix, 256 × 246; slice thickness, 1.0 mm; flip angle, 9°; and voxel size, 1 × 1 × 1 mm^3^. Conventional two-dimensional T1-weighted and T2-weighted images were obtained to detect any incidental findings. Diffusion tensor imaging (DTI) was performed with the following parameters: TR, 11,600 ms; TE, 107 ms; average, 1; FOV, 23 × 23 cm^2^; matrix, 128 × 128; reconstructed voxel size, 1.8 mm × 1.8 mm × 3 mm; slice thickness, 3 mm; flip angle, 90°; Apart from the one without diffusion weighting (b = 0 s/mm^2^), DTI was sequentially acquired in 30 non-collinear directions with a b value of 1000 s/mm^2^.

### FreeSurfer Analysis

**Figure 2:**
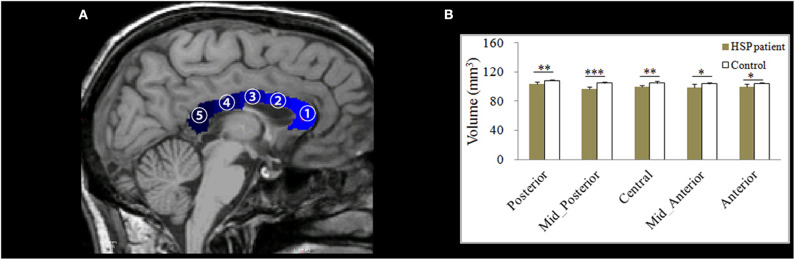
**(A)** An example of the division of the corpus callosum (shown in blue) into five segments on a T1-weighted image. **(B)** Differences in the mean volumes of the five subdivisions of the corpus callosum. ^*^p < 0.05; ^**^p < 0.01; ^***^p < 0.001.

FreeSurfer v6.0 (http://surfer.nmr.mgh.harvard.edu/) was used to analyze global gray- and white-matter volumes, volumes of the corpus callosum subdivisions, and cortical thickness. The processing stream has been described in our previous study (20). For each subject, the processing stream included motion correction, skull stripping, transformation to the Talairach space, and segmentation of gray/white matter tissue. The thickness measurements across the cortex were computed by finding the point on the gray matter–white matter boundary surface that was closest to a given point on the estimated pial surface, and then, averaging these two values. To map each subject to a common space, the surface that represented the gray/white matter border was registered to an average cortical surface atlas by using a non-linear procedure. After preprocessing, all steps were visually inspected to ensure accuracy. Errors were fixed by manual intervention following standard procedures (https://surfer.nmr.mgh.harvard.edu/fswiki/FsTutorial/TroubleshootingData#Fixingerrors). Group comparisons were performed using a vertex-by-vertex general linear model, with age, gender, and education as covariates. For this analysis, the thickness maps for all subjects were converted to the common atlas space. The data were spatially smoothed using a Gaussian kernel of 10 mm full-width at half-maximum. All results were corrected for multiple comparisons by using the permutation test at a vertex-wise/cluster-forming threshold of *p* < 0.0001 and a cluster-wise threshold of *p* < 0.05, and adjusting the *p*-values for the two hemispheres. To correct for multiple comparisons, a precomputed Z Monte Carlo simulation with 10,000 iterations was performed. This study only reported the cluster with a continuous extent of 100 mm^2^. On T1-weighted images, the corpus callosum was automatically segmented into anterior, mid-anterior, central, mid-posterior, and posterior subdivisions (Figure 2A).

### FIRST Analysis

FIRST v5.1.0 (http://www.fmrib.ox.ac.uk/fsl/) was used to segment and calculate subcortical structures. The data analysis was performed according to the method described by Patenaude et al. (21). The FIRST data-processing pipeline begins with two-stage affine registration to the MNI template (resolution, 1 mm; degrees of freedom, 12). The entire brain was registered to the template; a mask was used to refine the registration for subcortical structures. The caudate, thalamus, putamen, pallidum, and hippocampus were segmented. To minimize partial volume effects, ventricle masks were calculated using SIENA (http://www.fmrib.ox.ac.uk/fsl/) and applied to the subcortical segmentation. The FIRST segmentation results were validated using expert manual delineation. The FIRST segmentation results were validated by two expert neurologists, and in one set the automated results are quantitatively and qualitatively compared with expert manual delineation. Adjusting for intracranial volume was conducted before between-group comparisons.

### Tract-Based Spatial Statistics (TBSS) Analysis

Diffusion-tensor images were analyzed using FSL v5.1.0. The processing stream has been described in our previous study (20), and included eddy current and head movement corrections, binary brain mask preparation, fractional anisotropy (FA), mean diffusivity (MD), axial diffusivity (AD), and radial diffusivity (RD) maps generation with the FMRIB's Diffusion Toolbox Diffusion Toolbox (FDT-FMRIB), and map analysis with TBSS (FMRIB Software Library). To create an FA skeleton, we aligned the FA images to a template that was chosen arbitrarily from the FA images through non-linear registrations. The aligned images were transformed to the MNI 152 space. Individual FA images were projected onto the mean FA skeleton, and thresholded to an FA value of ≥0.2. Finally, voxel-wise statistical analysis of the FA images was conducted. The MD, AD, and RD images were analyzed using the FA images to achieve the non-linear registration and skeletonization stages as well as to estimate the MD, AD, and RD images from each individual subject onto the mean FA skeleton. Finally, cross-subject, voxel-wise, statistical analyses of FA, MD, AD, and RD were carried out. Threshold-free cluster enhancement was examined to correct for multiple comparisons by using the family-wise error correction at *p* < 0.05 with 5,000 permutations.

### Statistical Analysis

We used the Shapiro-Wilk test to check for the normality of the data on the total MoCA score and its subscores. Data with a normal distribution were presented as the mean ± standard deviation and compared using the Student *t*-test. Non-normally distributed data were expressed as median (interquartile range), and non-parametric Wilcoxon rank sum tests were used for comparisons between groups. Independent samples *t*-tests were used for between-group comparisons of MRI measurements, with the level of significance set at *p* < 0.05. Correction for false discovery rate and threshold-free cluster enhancement for multiple comparisons across space were conducted, with gender, age, and education as covariates. Partial correlation was used to assess the correlations of all MRI measurements with disease duration, SPRS score, and MoCA score, with gender and age as covariates. Dunn-Sidak correction was applied to adjust for multiple comparisons.

## Results

### Clinical Information

The age of onset and symptom severity greatly differed between the patients, even though they were all members of a single family and carried identical mutations. The average age at symptom onset was 34.7 ± 11.1 years (range, 23–55 years). The main neurological findings were spasticity (6/9), weakness (9/9), amyotrophy (4/9), and dystonia (4/9) (Table 1).

### Neuropsychological Examination

The MoCA scores showed that most patients (7/9) had mild cognitive impairment, mainly in the visuospatial and executive functioning domains, as well as delayed recall (Table 2). Compared to the healthy controls, the patients had lower MoCA scores (*p* < 0.01), lower subscores in the visuospatial and executive functioning domains (*p* < 0.05), and delayed recall (*p* < 0.05; Table 3). The MoCA score significantly correlated with disease duration (r = −0.901, *p* = 0.006).

**Table 2:** Montreal cognitive assessment (MoCA) scores of the HSP patients.

	**II:1**	**II:7**	**II:9**	**II:11**	**III:1**	**III:5**	**III:7**	**III:8**	**III:9**
Education (years)	3	13	7	0	15	15	6	12	10
Visuospatial and executive functioning	0	1	1	0	0	0	1	1	2
Animal naming	3	3	3	3	3	3	3	3	3
Attention	5	6	5	4	6	5	6	6	6
Language	3	3	3	3	3	3	3	3	3
Abstraction	2	2	2	2	2	2	2	2	2
Delayed recall	0	4	3	4	3	4	2	4	3
Orientation	6	6	6	6	4	6	6	6	6
MoCA score	20	25	24	23	21	23	24	26	26

*Education level: 1 point is added to the test-taker's score if the subject has 12 years or less of formal education*.

*Patients II 7 and III 5 were unable to undergo MRI scanning because they had metal implants in their body*.

**Table 3:** MoCA scores of control subjects and HSP patients.

	**HSP patients**	**Controls**	**t/Z**	***P***
Level of education (years)	7.57 ± 5.19	8.29 ± 4.96	*t* = −0.263	0.797
Visuospatial and executive functioning	1.00 ± 1.00	5.00 ± 0.00	−3.290	0.001
Animal naming	3.00 ± 0.00	3.00 ± 0.00		
Attention	6.00 ± 1.00	6.00 ± 1.00	−0.680	0.496
Language	3.00 ± 0.00	3.00 ± 0.00		
Abstraction	2.00 ± 0.00	2.00 ± 0.00	−1	0.317
Delayed recall	3.00 ± 2.00	5.00 ± 1.00	−2.989	0.003
Orientation	6.00 ± 0.00	6.00 ± 1.00	−0.964	0.335
MoCA total score	23.43 ± 2.30	29.29 ± 0.76	*t* = −6.403	<0.001

*HSP, hereditary spastic paraplegia; MoCA, Montreal Cognitive Assessment*.

### Identification of the *SPAST* c.1321+2T>C Mutation

**Figure 3:**
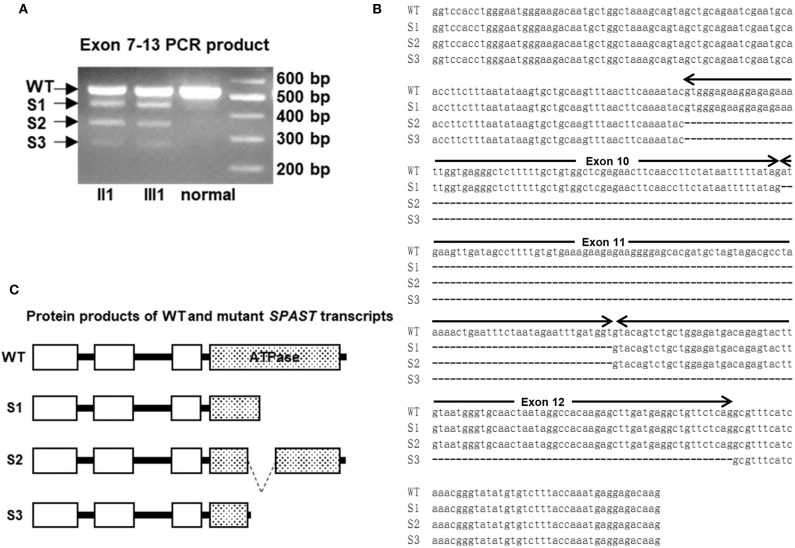
**(A)** The cDNAs from two patients and a healthy control subject were amplified with primers for exons 8–13 of *SPAST*. The longest fragment corresponds to the wild-type (WT) transcript, while the other three shorter fragments are the results of exon skipping. **(B)** Sequence alignment of the WT and c.1321+2T>C mutant *SPAST* transcripts. **(C)** Schematic illustration of the protein products of the mutant transcripts of *SPAST* resulted from c.1321+2T>C mutation.

A novel heterozygous mutation in the *SPAST* gene (NM_014946.3, c.1321+2T>C) was detected in patients II 1, II 7, II 9, II 11, III 1, III 5, III 7, III 8, and III 9 (Figure 1B). This mutation is located in intron 10 near the splicing site. We failed to detect any known HSP-associated mutations in the *SPAST, ATL1*, and *REEP1* genes in this family (22–24). We also excluded the potential effects of 50 genes associated with HSP (16). HSF3 analysis with the HSF Matrices and MaxEnt algorithms indicated a broken wild-type donor site. Alamut analysis predicted exon-10 skipping. To experimentally confirm the effect of the c.1321+2T>C mutation on the mRNA transcripts of *SPAST*, we amplified and analyzed the *SPAST* transcripts by using the patients' blood samples. Compared to the wild-type *SPAST* transcript, three mutant transcripts with shorter and varied lengths were detected in the patients' samples (Figure 3A). Different from the Alamut prediction, exon 11 was missing in the longest mutant transcript (defined as splicing mutant 1, S1), exons 10 and 11 were missing in the median length mutant (splicing mutant 2, S2), and exons 10, 11, and 12 were missing in the shortest mutant (splicing mutant 3, S3; Figure 3B). From the sequences of these PCR products, it was predicted that the S1, S2, and S3 mutants will produce protein products with varied portions of the *SPAST* ATPase domain deleted (Figure 3C).

### Total Gray-Matter and White-Matter Volumes

Both the gray-matter volume (602,436 ± 27,322 vs. 797,128 ± 47,968 mm^3^) and white-matter volume (526,713 ± 58,084 vs. 630,121 ± 17,784 mm^3^) were significantly lower in the HSP patients than in controls (both, *p* < 0.001).

### Volumes of Corpus Callosum Subdivisions

The volumes of all corpus callosum subdivisions were significantly lower in the HSP patients than in the controls (anterior: *p* = 0.014, mid-anterior: *p* = 0.033, central: *p* = 0.002, mid-posterior: *p* < 0.001, and posterior: *p* = 0.004, respectively; Figure 2B).

### Cortical Thickness

**Figure 4:**
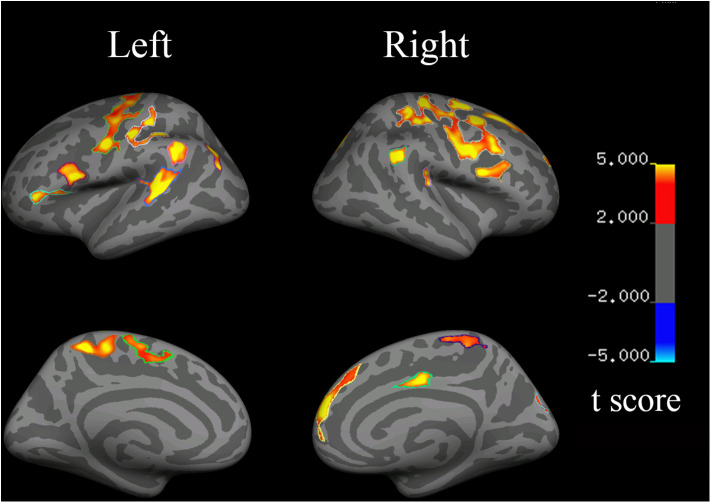
Changes in cortical thickness in HSP patients (vs. healthy controls). Red to yellow indicates a decrease in cortical thickness.

Compared with the controls, the HSP patients showed significantly decreased cortical thickness (>200 mm^2^) in the bilateral superior frontal gyri, inferior precentral cortices, central cortices, and inferior parietal cortices; the left inferior frontal triangular cortex, postcentral gyrus, and superior temporal gyrus; and the right middle cingulate cortex and superior occipital cortex (Figure 4, Table 4).

**Table 4:** Regional information of changed cortical thickness in HSP patients.

**Areas**	**Size (mm^**2**^)**	**Cortical thickness (mm)**		**MNI coordinate**			***t* (peak)**
		**Patient**	**Control**	**x**	**y**	**z**	
**Left**							
Superior frontal gyrus	469	1.8 (0.1)	2.3 (0.3)	−8	−14	58	4.334
Inferior precentral cortex	497	2.0 (0.2)	2.6 (0.3)	−46	10	16	5.974
Inferior frontal triangular cortex	428	2.4 (0.1)	2.9 (0.2)	−46	35	1	5.358
Central cortex	1995	1.9 (0.2)	2.6 (0.2)	−49	−8	34	6.466
Postcentral gyrus	986	1.8 (0.1)	2.4 (0.2)	−33	−33	49	6.612
Inferior parietal cortex	467	2.1 (0.1)	2.8 (0.3)	−32	−79	34	6.382
Superior temporal gyrus	1,029	2.1 (0.1)	2.7 (0.2)	−65	−37	7	7.704
**Right**							
Superior frontal gyrus	1,011	2.4 (0.2)	3.1 (0.3)	9	53	26	6.325
Superior frontal gyrus	412	2.0 (0.2)	2.5 (0.3)	22	19	51	6.039
Inferior precentral cortex	447	1.7 (0.1)	2.2 (0.3)	47	5	17	6.129
Central cortex	4,144	1.9 (0.1)	2.5 (0.1)	22	−29	52	8.128
Inferior parietal cortex	387	2.2 (0.1)	2.9 (0.2)	58	−41	29	6.916
Middle cingulate cortex	230	2.2 (0.2)	2.8 (0.1)	5	−9	39	5.838
Superior occipital cortex	501	2.4 (0.2)	2.9 (0.2)	25	−74	37	5.275

*HSP, hereditary spastic paraplegia*.

Disease duration was negatively correlated with the cortical thickness in the left precentral cortex (r = −0.782, *p* = 0.033), left inferior frontal cortex (r = −0.853, *p* = 0.007), right temporal cortex (r = −0.753, *p* = 0.025), right precentral cortex (r = −0.792, *p* = 0.017), and right frontal cortex (r = −0.680, *p* = 0.046). The cortical thickness in the right inferior frontal cortex was negatively correlated with the SPRS score (r = −0.785, *p* = 0.032).

The visuospatial and executive functioning score correlated with the cortical thickness in the left inferior precentral cortex (r = 0.693, *p* = 0.042) and superior temporal gyrus (r = 0.750, *p* = 0.026), and the right inferior precentral cortex (r = 0.723, *p* = 0.033) and inferior parietal cortex (r = 0.846, *p* = 0.008). The delayed recall score correlated with the cortical thickness in the left inferior precentral cortex (r = 0.762, *p* = 0.023), inferior frontal triangular cortex (r = 0.752, *p* = 0.026), and superior temporal gyrus (r = 0.733, *p* = 0.030), and the right superior frontal gyrus (r = 0.695, *p* = 0.042), inferior parietal cortex (r = 0.733, *p* = 0.031), and middle cingulate cortex (r = 0.733, *p* = 0.030).

### Volume of Subcortical Gray Matter

**Figure 5:**
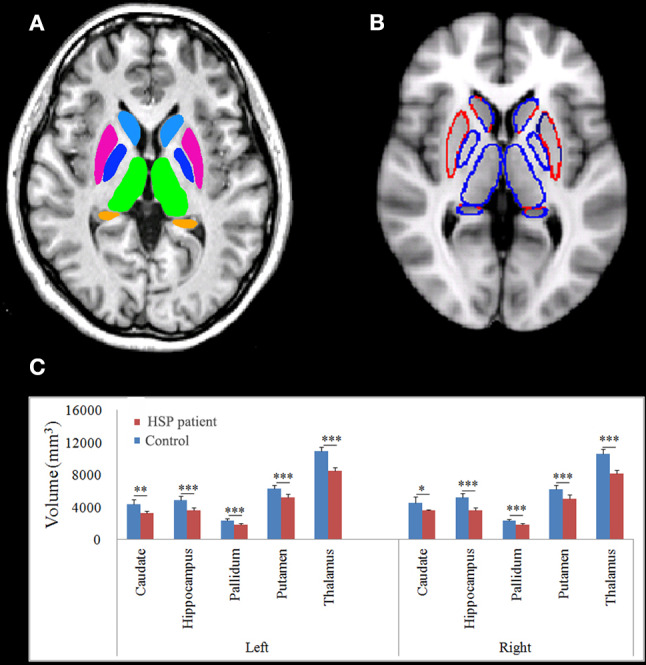
Normalized subcortical structural volume estimated using FIRST. **(A)** Colors are only for illustration of the estimated subcortical structural volume. **(B)** The blue line indicates decreased volume. **(C)** Differences in the mean volumes of the subcortical structures. ^*^p < 0.05; ^**^p < 0.01; ^***^p < 0.001.

The thalamus, caudate, pallidum, putamen, and hippocampus were segmented as shown in Figure 5. HSP patients showed significantly lower volumes than healthy controls in the left caudate (*p* = 0.001), hippocampus (*p* < 0.001), pallidum (*p* < 0.001), putamen (*p* < 0.001), and thalamus (*p* < 0.001) and the right caudate (*p* = 0.011), pallidum (*p* < 0.001), putamen (*p* < 0.001), and thalamus (*p* < 0.001).

Disease duration negatively correlated with the volume in the left hippocampus (r = −0.899, *p* = 0.007), pallidum (r = −0.742, *p* = 0.046), putamen (r = −0.933, *p* = 0.003), and thalamus (r = −0.870, *p* = 0.012) and the right caudate (r = −793, *p* = 0.030), pallidum (r = −0.773, *p* = 0.036), putamen (r = −0.825, *p* = 0.022), and thalamus (r = −0.748, *p* = 0.044). The volumes of the subcortical nuclei were negatively but not significantly correlated with SPRS scores. The volume in the right hippocampus correlated with the visuospatial and executive functioning score (r = 0.733, *p* = 0.049).

### FA, MD, AD, and RD Values of White Matter

**Figure 6:**
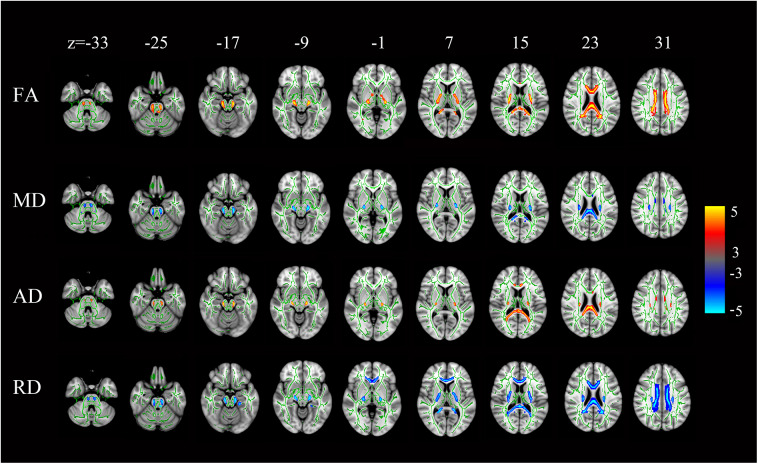
Statistical maps of the comparison of FA, MD, AD, and RD values on a voxel-wise basis. HSP patients (vs. controls) showed significantly lower FA and AD values and significantly higher MD and RD values in the corpus callosum and the corticospinal tract neurons from the motor and premotor cortices to the bulb and throughout the posterior limb of the internal capsule. All levels, *p* < 0.001, corrected. Red to yellow indicates a decrease in FA and AD values; blue indicates an increase in MD and RD values.

Compared with the controls, the HSP patients showed significantly lower FA and AD values and significantly higher MD and RD values in the splenium, isthmus, and body of the corpus callosum (all, *p* < 0.001) and in the corticospinal tract, deriving from the motor and premotor cortices and going through the posterior limb of the internal capsule (all, *p* < 0.001; Figure 6).

The FA, AD, MD, and RD values of the white matter were not correlated with disease duration, SPRS scores, and MoCA scores, in the corpus callosum or corticospinal tract.

## Discussion

A novel heterozygous mutation in the *SPAST* gene in a Chinese family was linked to cognitive impairment and spastic paraparesis. The patients presented with decreased motor and premotor cortical thickness, atrophic subcortical gray matter, and corpus callosum and corticospinal tract white-matter damage. Right hippocampal cortical thickness and gray-matter volume positively correlated with cognitive measurements. Subcortical gray-matter volume, cortical thickness, and cognitive measurements negatively correlated with disease duration. Right inferior frontal cortex thickness, but not subcortical gray-matter volume and white-matter FA, correlated with SPRS scores.

Cortical thinning was more widespread than previously thought, which is consistent with decreased gray-matter volumes in the mediodorsal thalamus, mid-cingulate cortex, and cerebellum found on voxel-based morphometry (VBM) in SPG4-HSP patients (14). To measure cortical thickness, we used FreeSurfer, an automatic segmentation-based post-processing algorithm with advantages over VBM (25). Our findings are consistent with those of Faber et al. (26), who found decreased cortical thickness in the precentral and paracentral motor cortices, superior parietal cortices, superior temporal gyrus, and cingulate cortices in SPG11-HSP, which suggests that SPG4-HSP and SPG11-HSP have similar cortical mechanisms. However, Rezende et al. (15) did not find cortical thinning in SPG4-HSP on FreeSurfer analysis. This difference in the results of their study and our study may be attributable to the fact that our patients were recruited from a family. Moreover, their patients had a broader age range than our patients.

Decreased thalamus and basal ganglia volumes have been described in patients with distinct HSP mutations. VBM has shown reduced thalamic subnuclei volume in SPG4-HSP (14). VBM also showed significantly decreased gray-matter volume in the thalamus and lentiform nuclei in SPG11-HSP (27). Using the T1-MultiAtlas tool, Faber et al. (26) found significantly decreased thalamus, accumbens nucleus, putamen, substantia nigra, amygdala, and red nucleus volumes in SPG11-HSP. These findings are consistent with our results of FIRST analysis, which demonstrated extensive deep gray-matter abnormality.

On TBSS analysis, we identified limited decreases in white-matter FA and AD in the upper portion of corticospinal tracts and the entire corpus callosum. TBSS has been used for white-matter analysis in SPG4-HSP patients. Thin corpus callosum has been found in most subtypes of HSP, including SPG4, SPG7, SPG11, SPG15, SPG18, SPG21, SPG35, SPG46, SPG47, SPG49, SPG50, and SPG54 (28). Rezende et al. (15) identified decreased FA in the splenium, corticospinal tracts, and cerebral white-matter fiber tracts. Similarly, Lindig et al. (14) found decreased FA in the fornix, chiasma opticum, and frontal white-matter tracts. However, Garaci et al. (3) reported that the decreased FA was limited to the corpus callosum, and that white-matter changes were more severe and extensive in SPG11-HSP than in SPG4-HSP. In addition, using voxel-based analysis, Duning et al. (13) revealed significant decreases in FA values that were confined to the corticospinal tracts and temporal lobes in SPG4-HSP. In our study, cognitive function, cortical thickness, and subcortical gray-matter volume negatively correlated with disease duration, suggesting progressive cognitive dysfunction and gray-matter damage. Murphy et al. (17) also found evidence of progressive cognitive impairment in SPG4-HSP. In contrast, white-matter changes did not correlate with disease duration. Consistent with our findings, disease duration correlates with gray-matter but not white-matter changes in SPG11-HSP (26). However, three studies on SPG4-HSP have reported that white-matter changes negatively correlated with disease duration (13–15). Our results suggest that gray matter may be related with disease progression in SPG4-HSP.

Right inferior frontal cortex thickness negatively correlated with SPRS score. Several studies support that the frontal cortex is involved in spastic paraplegia. Frontal lobe and thalamic hypoperfusion has been reported in a family with HSP (29). Spastic paraplegia patients show modified neuronal activation of the contralateral inferior premotor and sensorimotor cortices during ankle and shoulder movements (30). Patients with homocarnosinosis and spastic paraplegia show extremely high frontal-cortical homocarnosine levels (31). Seipin, a glycoprotein associated with dominant hereditary motor neuron diseases, has been detected in the spinal cord and frontal cortex (32). In contrast to our findings, Rezende et al. (15) did not find any correlation between clinical features and gray-matter changes in SPG4-HSP. Lindig et al. (14) found that the corpus callosum white-matter FA correlated with SPRS score; however, no such correlation was found in our study, which may be due to the small sample size of our study. Our results suggest that cortical gray-matter changes may be associated with disease severity in SPG4-HSP.

Lower visuospatial and executive scores positively correlated with cortical thickness in the inferior parietal cortex, superior temporal gyrus, superior frontal gyrus, and inferior precentral cortex, which are located in the visuospatial attention network (33). Right hippocampal volume positively correlated with visuospatial and executive score, which is consistent with a previous review (34).

The limitation of our study is that the number of cases was small. Moreover, since all patients came from the same family and shared the same variant, it is possible that the structural characteristic herein reported does not reflect the overall scenario for HSP-SPG4, but rather the scenario for this specific variant. Finally, MoCA is a screening test, so the correlations made with each cognitive domain were limited.

Our MRI investigation of SPG4-HSP with a novel *SPAST* gene mutation suggests that: [1] brain damage is not restricted to white matter, but is widespread, [2] cortical gray-matter changes may be associated with motor deficits, and [3] cortical gray matter may be involved in cognition impairment in patients with SPG4.

## Data Availability Statement

The datasets analyzed in this study can be obtained from the NCBI SRA database, with the following accession no: PRJNA623078.

## Ethics Statement

The studies involving human participants were reviewed and approved by The study protocol was approved by the Research Committee and the institutional review board of the First Affiliated Hospital of Xiamen University. The patients/participants provided their written informed consent to participate in this study.

## Author Contributions

The clinical data was collected by YZ. YZ and JZ designed most of the investigation, data analysis, and wrote the manuscript. JL, HZ, and QM performed most of the investigation. CW, LF, HW, and DW contributed to interpretation of the data and analyses. All of the authors have read and approved the manuscript.

## Conflict of Interest

The authors declare that the research was conducted in the absence of any commercial or financial relationships that could be construed as a potential conflict of interest.

## Abbreviations

HSP: hereditary spastic paraplegia
FA: fractional anisotropy
SPRS: Spastic Paraplegia Rating Scale
MoCA: Montreal Cognitive Assessment
TR: repetition time
TE: echo time
FOV: field of view
DTI: diffusion tensor imaging
TBSS: tract-based spatial statistics
VBM: voxel-based morphometry
MD: mean diffusivity
AD: axial diffusivity
RD: radial diffusivity.
